# Single-center experience of endovascular treatment for patients with progressive posterior circulation cerebral infarction exceeding 24 h

**DOI:** 10.2478/abm-2023-0046

**Published:** 2023-09-17

**Authors:** Guangfeng Zhao, Xiongjun He, Yajie Liu, Liang Zhang, Kaifeng Li

**Affiliations:** Department of Encephalopathy, Hainan Province Hospital of Traditional Chinese Medicine, Haikou, Hainan 570203, China; Department of Neurology, Shenzhen Hospital of Southern Medical University, Shenzhen, Guangdong 518101, China

**Keywords:** endovascular treatment, posterior circulation, progressive cerebral infarction

## Abstract

**Background:**

Evidence of endovascular treatment (ET) for patients with progressive infarction of the posterior circulation exceeding 24 h is lacking.

**Objective:**

To evaluate the efficacy and safety of ET for progressive posterior circulation cerebral infarction.

**Methods:**

This retrospective study evaluated the ET for 18 patients with posterior circulation infarction caused by vertebrobasilar artery occlusion from July 2017 to November 2018. The conditions of patients worsened despite receiving intravenous thrombolysis or combination therapy with clopidogrel and aspirin. The time from the onset of cerebral infarction to puncture was >24 h. The preoperative National Institutes of Health Stroke Scale (NIHSS), modified Rankin Scale (mRS), and related risk factors of patients at 3 months were analyzed postoperatively.

**Results:**

The preoperative NIHSS score was 10.6 (IQR: 6.5), and the time from onset to puncture was 163.5 ± 144.7 h. Postoperative blood flow was modified thrombolysis in cerebral infarction (mTICI) grade 2b or above. During the follow-up period, 1 patient died of basilar artery re-occlusion and pulmonary infection, and 1 died of postoperative hyperperfusion hemorrhage, with a mortality rate of 11.1% (2/18). No recurrent ischemic events were observed in any of the 16 patients during the 3-month follow-up period. The mean mRS score was 1.3 (IQR: 2.3), and 75% patients (12/16) had an mRS score of 0–2. There were no significant differences in age, gender, clinical characteristics, and stroke subtype between patients with mRS scores ≤2 and >2.

**Conclusion:**

In patients with progressive posterior circulation cerebral infarction caused by vertebral basilar artery occlusion, ET is effective and safe even if the time from onset to puncture exceeds 24 h.

The clinical manifestations of posterior circulation infarction vary significantly, which include dizziness, decreased consciousness, diplopia, visual field defects, and other symptoms that are different from typical symptoms of stroke. Patients with posterior circulation infarction often have a lower National Institutes of Health Stroke Scale (NIHSS) score than those with anterior circulation stroke, as well as fewer hemiplegic symptoms, less dysarthria, and fewer cognitive symptoms; therefore, these patients are prone to be misdiagnosed in the emergency department [1, 2]. Timely diagnosis of posterior circulation stroke or transient ischemic attack can avoid disabilities and reduce mortality. Delays in emergent treatment or secondary prevention or delayed or incorrect diagnosis may induce devastating consequences, such as potentially preventable death or severe disabilities [3]. At present, there is no consensus on the treatment of patients who lack early reperfusion; moreover, the Basilar Artery International Cooperation Study (BASICS) reports that intra-arterial therapy is not beneficial compared to drug therapy [4]. It is worth noting that the BASICS does not use modern mechanical thrombectomy devices that can improve reperfusion and clinical outcomes [5, 6]. Endovascular treatment (ET) is an important method for treating posterior circulation infarction. Meta-analysis by the Highly Effective Reperfusion Using Multiple Endovascular Devices (HERMES) group including 5 positive randomized controlled trials (MR CLEAN, ESCAPE, REVASCAT, SWIFT PRIME, and EXTEND-IA) confirmed the efficiency of mechanical thrombectomy for large vessel occlusion of the anterior circulation [7]. The DAWN (DWI or CTP Assessment with Clinical Mismatch in the Triage of Wake-Up and Late Presenting Strokes Undergoing Neurointervention with Trevo) and DEFUSE 3 (Endovascular Therapy Following Imaging Evaluation for Ischemic Stroke) trials extended the mechanical thrombectomy time window to 18–24 h [8, 9]. However, evidence of ET for patients with progressive infarction of the posterior circulation exceeding 24 h is lacking. Therefore, the current study aimed to evaluate the efficacy and safety of ET for progressive posterior circulation cerebral infarction.

## Methods

### Subjects

Patients with progressive cerebral infarction caused by large vessel occlusion or extremely severe stenosis of the posterior circulation who were treated in the Department of Neurology at the Shenzhen Hospital of Southern Medical University from July 2017 to November 2018 were enrolled in the study. The patients whose NIHSS score increased >2 points after hospitalization were defined as progressive cerebral infarction. The study protocol was approved by the Ethics Committee of the Shenzhen Hospital of Southern Medical University (certificate of approval no. KY2017-048-01). The written informed consent was obtained from the patients. We have used the Strengthening the Reporting of Observational studies in Epidemiology (STROBE) guidelines and The REporting of studies Conducted using Observational Routinely-collected health Data (RECORD) Statement [10, 11].

### Assessment

Magnetic resonance imaging (MRI) of the skull confirmed an acute cerebral infarction in the posterior circulation in all patients. Head and neck magnetic resonance angiography (MRA) or computed tomography angiography (CTA) identified large-vessel occlusion or extremely severe stenosis of the posterior circulation. Intraoperative angiography was performed to further determine and evaluate collateral circulation.

### Treatment

The treatment regimen followed the protocol of our previous study [12]. Briefly, large vessel occlusion of the posterior circulation was confirmed after angiography, and microguide wires and microcatheters were used to pass the lesion carefully to the distal vascular lumen. After the microcatheter was retracted, the vascular occlusion length was determined using micro-catheter angiography. A 1.5 mm gateway balloon was used to dilate the vascular lesion site, and then the reason of occlusion was analyzed by vascular morphology on microcatheter angiography. The vascular resistance during balloon dilatation, blood flow velocity, and blood flow volume with or without filling defect after balloon dilatation were measured. The operation was planned based on the medical history, risk factors, results of angiography, and resistance of microguide wires passing through the occlusion segment. If vascular stenosis was observed, a balloon of larger diameter was selected for expansion, and emergency stent implantation was performed when blood vessel dissection was found or the artery could not be recanalized after dilatation. Thrombectomy with retrievable stent placement was performed if thrombosis was suspected.

### Observation index

The preoperative NIHSS and modified Rankin Scale (mRS), postoperative complications, NIHSS and mRS at 3 months postoperatively. The patients were divided in to mRS score ≤2 and patients with mRS score >2, the related risk factors including age, gender, clinical characteristics, stroke subtype and occlusion site.

### Statistical analysis

SPSS 17.0 statistical software was used for data analysis. The measurement data were subjected to the normality tests (Kolmogorov–Smirnov test), and data with a normal distribution are expressed as the means ± standard deviation (SD); non-normally distributed measurement data are represented by the median and quartile values (M [P25, P75]). Count data are represented by the number of cases. Measurement data with uniform variance were analyzed using t tests, and those with nonuniform variance were analyzed using *t* tests. A Chi-square test was used to analyze count data. *P* < 0.05 was considered statistically significant.

## Results

### Basic characteristics

A total of 18 patients with progressive cerebral infarction caused by large vessel occlusion or extremely severe stenosis of the posterior circulation who were treated in the Department of Neurology at the Shenzhen Hospital of Southern Medical University from July 2017 to November 2018 were enrolled in the study.

The time from symptom onset to puncture ranged from 24.5 h to 3 weeks (average 163.5 h). The conditions of these patients were aggravated or relapsed repetitively after active drug treatment. The symptoms of 2 patients worsened after recombinant tissue plasminogen activator (rtPA) thrombolysis in other hospitals. The primary nervous symptoms and signs of the cerebral infarction progressively worsened, and NIHSS score increased to no less than 2 points [13].

Patients were classified according to the classification of the Trial of ORG 10172 in Acute Stroke Treatment (TOAST) etiology as follows: 15 cases of major atherosclerosis, 1 case of cardiac embolism, and 2 cases of arterial dissection. Among them, 14 cases involved brain stem infarction lesions, 5 cases involved cerebellum infarction lesions, 4 cases involved occipital infarction lesions, and 2 cases involved thalamic infarction lesions. Twelve patients were male and 6 patients were female, their ages ranged from 39 years to 78 years (average age of 60.3 years). In addition, 14 patients (77.8%) had hypertension, 4 patients (22.2%) had diabetes, 2 patients (11.1%) had coronary heart disease, 2 patients (11.1%) had atrial fibrillation, 5 patients (27.8%) had dyslipidemia, and 5 patients (27.8%) had a history of smoking (**Table 1**).

**Table 1.** Characteristics of patients with posterior circulation ischemic stroke

**Characteristics**	**N (%)**
Age (y) (mean ± SD)	60.3 ±13.5
Gender	
Male	12 (66.7)
Female	6 (33.3)
Vascular risk factors	
Yes	16 (88.9)
No	2 (11.1)
Hypertension	14 (77.8)
Diabetes mellitus	6 (33.3)
Dyslipidaemia	5 (27.8)
History of stroke	3 (16.7)
Coronary atherosclerotic cardiomyopathy	2 (11.1)
Atrial fibrillation	2 (11.1)
Smoking	5 (27.8)
Clinical characteristics	
Collateral flow	
Yes	13 (72.2)
No	5 (27.8)
Stroke subtype (TOAST)	
Large-artery atherosclerosis	14 (77.7)
Cardioembolism	1 (5.6)
Stroke of undetermined etiology	1 (5.6)
Stroke of other determined etiology: Dissection	2 (11.1)
Occlusion site	
Basilar or Basilar and Vertebral artery	11 (61.1)
Vertebral artery	7 (38.9)
Baseline NIHSS (median, IQR)	10, 4.5–12
Day 90 mRS (median, IQR)	1.3, 2.3
Time from onset to puncture (median, IQR)	116.5, 48–205.75
Day 90 NIHSS (median, IQR)	4.6, 8

### Operation and clinical prognosis

In total, 11 cases demonstrated basilar artery stenosis or occlusion, including 4 cases complicated with unilateral vertebral artery stenosis or occlusion. Among the 11 cases, compensation of the basilar artery from the posterior communicating artery was found in 5 cases, that from the meningeal artery was found in 4 cases, and 2 cases had no collateral circulation compensation. Moreover, 3 cases demonstrated unilateral vertebral artery stenosis or occlusion without collateral circulation, and 4 cases demonstrated bilateral vertebral artery stenosis or occlusion, including 2 cases compensated by meningeal artery and 2 cases compensated by anterior spinal artery.

Three patients received balloon dilation due to large infarction areas in the brainstem (infarction lesions >1/3 of the same section level). A total of 9 patients underwent thrombectomy using a Solitaire AB/Trevo/Aperio thrombectomy stent. Among them, a Solitaire AB stent was released during operation in 5 patients, and 3 patients underwent balloon dilatation due to restenosis within stents because of thrombus. An intracranial balloon-expandable stent (Apollo) and a self-expanding stent (Winspan) were used in 1 patient due to a long segmental arterial lesion. Two peripheral SD stents were implanted in 1 patient due to long segmental vertebral artery dissection. Winspan stents were implanted in 7 patients, Apollo stents were implanted in 2 patients, and SD stents were implanted in 2 patients. The lesion arteries of all patients were recanalized after operation, and the modified thrombolysis in cerebral infarction (mTICI) [14] scale recovered to grade 3 in 14 patients and to grade 2b in 4 patients. Moderate to severe residual stenosis was found in 2 patients. Among these 2 patients, postoperative severe residual stenosis was found after stent implantation, and 50% residual stenosis was found even after expansion with a 2.5 mm balloon in 1 patient. The other patient underwent balloon dilatation due to a relatively large infarction area, and the residual stenosis was approximately 60%. Two patients underwent intra-arterial infusion of 10 mL of tirofiban solution during the operation, with intravenous pump infusion at 5 mL/h for 24 h after the operation. Residual stenosis of all the other patients was below 50% (**Table 2**).

**Table 2.** Operation and clinical prognosis

**Treatment and prognosis**	**N (%)**
Operation	
Balloon predilution	17 (94.4)
Thrombus extraction (Solitaire AB, Trevo, Aperio)	9 (50.0)
Released stents	
Solitaire AB	5 (26.3)
Winspan	8 (42.1)
Apollo	3 (15.8)
SD	3 (15.8)
Successful recanalization	
Complete recanalization (mTICI, 3)	14 (77.8)
Part recanalization (mTICI, 2b)	4 (22.2)
Clinical symptoms	
Clinical symptom improvement	15 (83.3)
Invalid	1 (5.6)
Aggravation or death	2 (11.1)

In all, one patient died of basilar artery re-occlusion and pulmonary infection after the operation. One patient died of hemorrhage related to postoperative hyperperfusion. Furthermore, four patients had pulmonary infections, 2 of whom had preoperative NIHSS scores of 12 and 14, and their 3-month postoperative mRS scores were 3 and 5, respectively. The other 12 patients had no complications. The 16 patients who survived regularly took secondary preventive drugs (clopidogrel 75 mg/d, aspirin 100 mg/d, and Lipitor 20–60 mg/d). No recurrent cerebral ischemia events were observed in any of the 16 patients during the 3-month follow-up period. The mean mRS score was 1.3 (IQR: 2.3), and 75% patients (12/16) had an mRS score of 0–2. No aggravation of residual stenosis was identified.

### Risk factors

Patients with posterior circulation progressive infarction had an average time from symptom onset to puncture of 163.5 h (25–528 h). The postoperative blood flow of all patients restored to mTICI grade 2b or above, and the 90-d mRS scores of 75% of the 16 survivors (12/16) were 0–2. A total of four patients with a 90-d mRS score of >2 had basilar artery occlusion (BAO) or severe stenosis (*P* = 0.0417; **Table 3**), including 3 patients with vascular lesions in the middle segment of the basilar artery and 1 patient with a vascular lesion at the tip of the basilar artery. The postoperative mTICI scores were all grade 3, the preoperative NIHSS scores ranged from 12 to 17, and skull MRI showed multiple infarction lesions in all 4 patients, including 1 case of bilateral cerebral peduncle and pons infarction, 1 case of bilateral thalamic infarction, and 2 cases of bilateral pons infarction. There were no significant differences in age, gender, clinical characteristics, and stroke subtype between patients with mRS scores ≤2 and patients with mRS scores >2 (*P* > 0.05).

**Table 3.** Univariate analysis of clinical, radiological, and procedural variables affecting functional outcome 90 days after stroke

**Characteristics**	**mRS score ≤2**	**mRS score >2**	***P***
Mean age (years)	57.7 ± 15.4	61.5 ± 3.3	0.5640
Gender, male/female	9/3	2/2	0.3502
Clinical characteristics			
Baseline NIHSS (median, IQR)	7.5, 4–10.5	13, 10–15.5	0.1040
Time from onset to puncture	104, 58.3–288.8	132.5, 36.5–146.5	0.7159
Collateralization, yes/no	9/3	3/1	1.0000
Stroke subtype (TOAST)			
Atherothrombosis	9	4	
Embolic	1	0	
Dissection	2	0	1.0000
Occlusion site			
Basilar artery or basilar and vertebral artery	5	4	
Vertebral artery	7	0	0.0884

### Typical case

**Figure 1.**
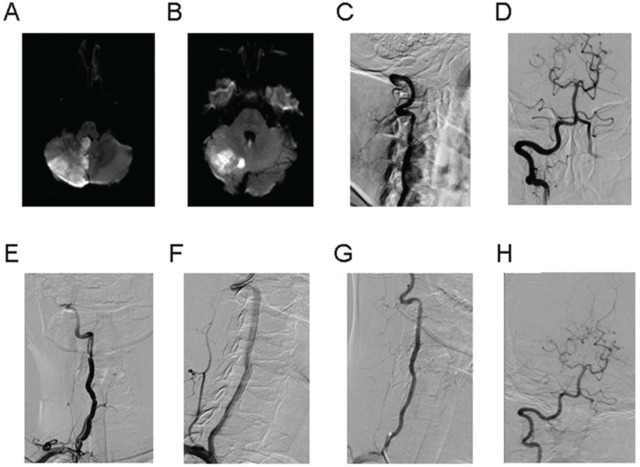
A typical case who underwent emergency stent implantation after thrombectomy. **(A, B)**. Skull MR showed subacute cerebral infarction in the right cerebellar hemisphere, cerebellar tonsil, and cerebellar vermis; **(C)**. Angiography showed occlusion of the V2 segment of the right vertebral artery far away; **(D)**. Thrombectomy was performed twice using an Aperio thrombectomy stent. The blood flow of the right vertebral artery was restored to an mTICI score of grade 3 after the dark red thrombus was aspirated; **(E)**. When the guiding catheter was retracted to the right subclavian artery, angiography revealed blood vessel dissection in the V2 segment of the vertebral artery and severe stenosis in the local lumen of the V3 segment; **(F)**. One SD stent (5*19 mm, Boston Science) was implanted in the dissection site of the V2 segment of the right vertebral artery, and 1 SD stent (4*19 mm, Boston Science) was implanted in the V3 segment of the right vertebral artery. The stent completely covered the vascular dissection with satisfactory angioplasty and an mTICI score of grade 3 of forward blood flow, without residual stenosis of the lumen; **(G, H)**. Follow-up angiography after 3 months showed no stenosis in the stents, and the intracranial blood supply was good. mTICI, modified thrombolysis in cerebral infarction; SD, standard deviation.

In this article, we present a typical case who underwent emergency stent implantation after thrombectomy. The case was a 39-year-old woman admitted to the hospital due to “dizziness for 3 d.” Her physical examination showed a positive Romberg sign, and the skull MRI confirmed posterior circulation infarction attributed to occlusion of the V2 segment of the right vertebral artery. Thrombus was removed after thrombectomy with retrievable stent placement performed twice. Then, stents were implanted in the V2 segment because of dissection and the V3 segment because of severe stenosis. There was no residual stenosis after operation and no stenosis in stents after 3 months (**Figure 1**).

## Discussion

The prognosis of patients with BAO remains poor, especially in the absence of early reperfusion [15]. The BASICS and another study in Germany showed that the functional outcome was poor in patients with severe clinical conditions in whom recanalization was achieved beyond 9 h [4, 16]. Evidence from a systematic analysis on the outcome of BAO after intravenous or intra-arterial thrombolysis showed that only 2% of 420 patients had a good outcome in the absence of basilar artery recanalization [17]. Severe disabilities (such as atresia syndrome) are common among survivors without recanalization. BASICS [18] was a randomized controlled, multicenter, open-label phase III intervention trial investigating the efficacy and safety of additional intra-arterial treatment (IAT) after intravenous thrombolysis (IVT) in patients with BAO. The primary outcome was favorable outcome at day 90 defined as a mRS score of 0–3. However, the 10th interim analysis conducted in February 2019 failed to achieve positive results (http://basicstrial.com/Main.html). Recently published Basilar Artery Occlusion Endovascular Intervention versus Standard Medical Treatment (BEST) study [19] was a multicenter, randomized, open-label trial assessing patients presenting within 8 h of vertebrobasilar (VB) occlusion, which aimed to evaluate the safety and efficacy of ET of acute stroke caused by VB artery occlusion. The trial was terminated after 131 patients had been randomly assigned due to the high crossover rate and poor recruitment. In the intention-to-treat analysis, there was no difference in the proportion of participants with of mRSs 0–3 at 90 d [19]. Secondary prespecified analysis of the primary outcome showed higher rates of mRS 0–3 at 90 d in patients who received the intervention than in those who received standard medical therapy alone in both per-protocol and as-treated populations [19]. It is worth noting that atherosclerotic stenosis accounted for 53% of the population in the BEST study [19].

In our study, the postoperative mTICI scores were all grade 3, the preoperative NIHSS scores ranged from 12 to 17, and skull MRI showed multiple infarction lesions in all 4 patients, including 1 case of bilateral cerebral peduncle and pons infarction, 1 case of bilateral thalamic infarction, and 2 cases of bilateral pons infarction. The most common causes of posterior circulation stroke include vertebral basilar artery atherosclerotic occlusion or embolism, dissection, and cardiogenic emboli [20, 21]. The Chinese Intracranial Atherosclerosis (CICAS) study in 2014 showed that the incidence of intracranial atherosclerosis was 46.6% in Chinese patients with ischemic stroke or transient ischemic attack [22] compared to only 8%–10% in the North American population [23, 24]. Annual stroke risk was 19% in patients with 70%–99% stenosis, and 23.4% in those with symptomatic intracranial chronic arterial occlusion [25]. A German study investigated the distribution of steno-occlusive disease and the associated rate of recurrence in 4157 patients with acute cerebral ischemia. A total of 48 patients (1.2%) with BAO were included. Symptomatic vessel occlusions were associated with a higher mortality rate and stroke recurrence rates than stenosis. The rate of recurrent stroke during the first 3 d in patients with BAO was 14.6%. The mortality rate within 100 d was 44.7%, the transient ischemic attack recurrence rate between day 4 and 1 year was 3%, and stroke recurrence rate was 9.1% in those patients with BAO [26]. Among the cases we analyzed, atherosclerotic stenosis or occlusion accounted for 83.3%, dissection accounted for 11.1%, and cardiogenic embolism accounted for 5.6% of cases. The preoperative NIHSS scores of patients with dissection and cardiogenic embolization ranged from 2 to 4, and the postoperative 90-d mRS scores were 0–1.

A previous study hypothesized that salvageable tissue in the territory of posterior circulation showed a higher time window for anterior circulation stroke thrombolysis, which may be due to the high proportion of white matter in the brainstem; this makes it more resistant to ischemia than other brain tissues, and better collaterals in the posterior circulation than in the anterior circulation, leading to slower progression of ischemic tissue damage [27]. We also noticed that collaterals were more abundant in posterior circulation by angiography. When proximal BAO occurred, the unilateral posterior inferior cerebellar artery could compensate the middle and distal segments of the basilar artery by the meningeal branch artery. The posterior inferior cerebellar artery could also compensate the ipsilateral anterior inferior cerebellar artery and the contralateral inferior cerebellar artery. When complete occlusion occurred in large vessels or thrombus in the occlusion segment still prolonged, blood flow to the distal branches decreased and the distal narrowed vessels failed to form effective collateral circulation to bypass the blockage due to low perfusion. A multicenter, prospective, blinded, longitudinal cohort study (Vertebrobasilar Flow Evaluation and Risk of Transient Ischemic Attack and Stroke, VERiTAS) [28] included 72 patients with recent VB transient ischemic attack or stroke and 50% or more atherosclerotic stenosis or occlusion in vertebral and/or basilar arteries who underwent large-vessel flow measurement in the VB territory using quantitative magnetic resonance angiography (QMRA). During the 24 month follow-up, distal flow status was low in 18 of the 72 participants (25%), which was significantly associated with risk of a subsequent VB stroke (*P* = 0.04). They proposed that the distal flow status was robustly associated with risk of a subsequent stroke in patients with symptomatic atherosclerotic VB occlusive disease. A total of nine patients in our study received thrombectomy stent treatment, and fresh thrombus was identified in 4 patients when retrieving the stents, of whom 2 patients exhibited atherosclerotic stenosis complicated with thrombus, with 90-d mRS scores of ≥3, 1 patient exhibited dissection complicated with thrombus, and 1 patient exhibited cardiac embolism; the latter 2 patients had 90-d mRS scores of 0.

Our center has performed recanalization operations of intracranial and extracranial vascular occlusions for many years [29] and has extensive experience in operation and treatments of intraoperative and postoperative complications of recanalization procedures. Therefore, postoperative blood flow of all patients in this study was greater than mTICI grade 2b with low mortality (11.1%).

There were some limitations to this study. First, only 18 patients from a single center were retrospectively analyzed. Second, control groups were not conducted. It is expected that multicenter and high-quality clinical trials on posterior circulation infarction patients whose conditions are progressively aggravated beyond 24 h will be conducted in future.

In conclusion, in patients with progressive posterior circulation cerebral infarction caused by vertebral BAO, ET is effective and safe even if the time from onset to puncture exceeds 24 h.

## References

[1] Banerjee G, Stone SP, Werring DJ (2018). Posterior circulation ischaemic stroke. BMJ.

[2] Zürcher E, Richoz B, Faouzi M, Michel P (2019). Differences in ischemic anterior and posterior circulation strokes: a clinico-radiological and outcome analysis. J Stroke Cerebrovasc Dis.

[3] Kuruvilla A, Bhattacharya P, Rajamani K, Chaturvedi S (2011). Factors associated with misdiagnosis of acute stroke in young adults. J Stroke Cerebrovasc Dis.

[4] Schonewille WJ, Wijman CA, Michel P, Rueckert CM, Weimar C, Mattle HP (2009). Treatment and outcomes of acute basilar artery occlusion in the Basilar Artery International Cooperation Study (BASICS): a prospective registry study. Lancet Neurol.

[5] Nogueira RG, Lutsep HL, Gupta R, Jovin TG, Albers GW, Walker GA (2012). Trevo versus Merci retrievers for thrombectomy revascularisation of large vessel occlusions in acute ischaemic stroke (TREVO 2): a randomised trial. Lancet.

[6] Saver JL, Jahan R, Levy EI, Jovin TG, Baxter B, Nogueira RG (2012). Solitaire flow restoration device versus the Merci Retriever in patients with acute ischaemic stroke (SWIFT): a randomised, parallel-group, non-inferiority trial. Lancet.

[7] Goyal M, Menon BK, van Zwam WH, Dippel DW, Mitchell PJ, Demchuk AM (2016). Endovascular thrombectomy after large-vessel ischaemic stroke: a meta-analysis of individual patient data from five randomised trials. Lancet.

[8] Nogueira RG, Jadhav AP, Haussen DC, Bonafe A, Budzik RF, Bhuva P (2018). Thrombectomy 6 to 24 hours after stroke with a mismatch between deficit and infarct. N Engl J Med.

[9] Albers GW, Marks MP, Kemp S, Christensen S, Tsai JP, Ortega-Gutierrez S (2018). Thrombectomy for stroke at 6 to 16 hours with selection by perfusion imaging. N Engl J Med.

[10] von Elm E, Altman DG, Egger M, Pocock SJ, Gotzsche PC, Vandenbroucke JP (2014). The Strengthening the Reporting of Observational Studies in Epidemiology (STROBE) statement: guidelines for reporting observational studies. Int J Surg.

[11] Benchimol EI, Smeeth L, Guttmann A, Harron K, Hemkens LG, Moher D (2016). Das RECORD-Statement zum Berichten von Beobachtungsstudien, die routinemäßig gesammelte Gesundheitsdaten verwenden [The REporting of studies Conducted using Observational Routinely-collected health Data (RECORD) statement]. Z Evid Fortbild Qual Gesundhwes.

[12] He X, Zhang L, Yang J, Zheng H, Li K, Liu Y (2017). Multimodal therapy for non-superacute vertebral basilar artery occlusion. Interv Neurol.

[13] Philipps J, Thomalla G, Glahn J, Schwarze M, Rother J (2009). Treatment of progressive stroke with tirofiban – experience in 35 patients. Cerebrovasc Dis.

[14] Yoo AJ, Simonsen CZ, Prabhakaran S, Chaudhry ZA, Issa MA, Fugate JE (2013). Refining angiographic biomarkers of revascularization: improving outcome prediction after intra-arterial therapy. Stroke.

[15] Singer OC, Berkefeld J, Nolte CH, Bohner G, Haring HP, Trenkler J (2015). Mechanical recanalization in basilar artery occlusion: the ENDOSTROKE study. Ann Neurol.

[16] Ottomeyer C, Zeller J, Fesl G, Holtmannspötter M, Opherk C, Bender A (2012). Multimodal recanalization therapy in acute basilar artery occlusion: long-term functional outcome and quality of life. Stroke.

[17] Lindsberg PJ, Mattle HP (2006). Therapy of basilar artery occlusion: a systematic analysis comparing intra-arterial and intravenous thrombolysis. Stroke.

[18] van der Hoeven EJ, Schonewille WJ, Vos JA, Algra A, Audebert HJ, Berge E (2013). The Basilar Artery International Cooperation Study (BASICS): study protocol for a randomised controlled trial. Trials.

[19] Liu X, Dai Q, Ye R, Zi W, Liu Y, Wang H (2020). Endovascular treatment versus standard medical treatment for vertebrobasilar artery occlusion (BEST): an open-label, randomised controlled trial. Lancet Neurol.

[20] Savitz SI, Caplan LR (2005). Vertebrobasilar disease. N Engl J Med.

[21] Caplan LR, Wityk RJ, Glass TA, Tapia J, Pazdera L, Chang HM (2004). New England medical center posterior circulation registry. Ann Neurol.

[22] Wang Y, Zhao X, Liu L, Soo YO, Pu Y, Pan Y (2014). Prevalence and outcomes of symptomatic intracranial large artery stenoses and occlusions in China: the Chinese Intracranial Atherosclerosis (CICAS) Study. Stroke.

[23] Sacco RL, Kargman DE, Gu Q, Zamanillo MC (1995). Race-ethnicity and determinants of intracranial atherosclerotic cerebral infarction. The Northern Manhattan Stroke Study. Stroke.

[24] Wong LK (2006). Global burden of intracranial atherosclerosis. Int J Stroke.

[25] Wong KS, Huang YN, Gao S, Lam WW, Chan YL, Kay R (1998). Intracranial stenosis in Chinese patients with acute stroke. Neurology.

[26] Weimar C, Goertler M, Harms L, Diener HC (2006). Distribution and outcome of symptomatic stenoses and occlusions in patients with acute cerebral ischemia. Arch Neurol.

[27] Mattle HP, Arnold M, Lindsberg PJ, Schonewille WJ, Schroth G (2011). Basilar artery occlusion. Lancet Neurol.

[28] Amin-Hanjani S, Pandey DK, Rose-Finnell L, Du X, Richardson D, Thulborn KR (2016). Effect of hemodynamics on stroke risk in symptomatic atherosclerotic vertebrobasilar occlusive disease. JAMA Neurol.

[29] He X, Liu Y (2012). Short-term efficacy observation of interventional therapy for 9 cases with symptomatic intercarotid artery occlusion. J Chin (Electronic Edition).

